# Predicting Weight Loss Success After Gastric Sleeve Surgery: A Machine Learning-Based Approach

**DOI:** 10.3390/nu17081391

**Published:** 2025-04-21

**Authors:** Mónica Casas Domínguez, Isabel Herrena Montano, Juan José López Gómez, Beatriz Ramos Bachiller, Daniel Antonio de Luis Román, Isabel de la Torre Díez

**Affiliations:** 1 Department of Signal Theory and Communications, and Telematics Engineering, University of Valladolid, Paseo de Belén, 15, 47011 Valladolid, Spain; isabel.herrera.montano@uva.es (I.H.M.); isator@uva.es (I.d.l.T.D.); 2 Research Center for Endocrinology and Clinical Nutrition, University of Valladolid, 47003 Valladolid, Spain; juanjose.lopez.gomez@uva.es (J.J.L.G.); bramosb@saludcastillayleon.es (B.R.B.); 3 Endocrinology and Nutrition Department, Clinical University Hospital of Valladolid, 47003 Valladolid, Spain

**Keywords:** machine learning, sleeve, bariatric surgery, risk factors, obesity

## Abstract

Background/Objectives: Obesity is a global health issue, and in this context, bariatric surgery is considered the most effective treatment for severe cases. However, postoperative outcomes vary widely among individuals, driving the development of tools to predict body weight loss success. The main objective of this paper is to evaluate predictive variables for successful weight loss one year after Sleeve bariatric surgery, defining success as a weight loss exceeding 30%. Methods: A dataset of 94 cases was included in this study. Data were collected between 2013 and 2018 from the Nutrition Section of the Endocrinology and Nutrition Department in the Eastern Area of Valladolid, Spain. Machine learning algorithms applied included Random Forest, Multilayer Perceptron, XGBoost, Decision Tree, Logistic Regression, and Support Vector Machines (SVMs). Results: The SVM model demonstrated the best performance, attaining an accuracy of 88% and an area under the curve (AUC) of 0.76 with a 95% CI between 0.5238 and 0.9658. The main predictive variables identified were potassium (K), folic acid, alkaline phosphatase (ALP), height, transferrin, weight, body mass index (BMI), triglyceride (Tg), Beck Depression Test score, and insulin levels. Conclusions: In conclusion, this study highlights the potential of machine learning models, particularly Support Vector Machines (SVMs), in predicting successful weight loss after Sleeve bariatric surgery. The key predictive variables identified include biochemical markers, anthropometric measures, and psychological factors, emphasizing the multifactorial nature of postoperative weight loss outcomes.

## 1. Introduction

Obesity is a chronic and multifactorial disease that has significantly increased in prevalence worldwide. It has become a major public health issue, affecting approximately 36% of the adult population in the United States and continuing to rise in developing countries [1]. Beyond its impact on quality of life, obesity is associated with several comorbidities, including cardiovascular diseases, type 2 diabetes mellitus, and certain types of cancer [2]. In this context, bariatric surgery has emerged as the most effective treatment for severe and morbid obesity, offering sustained weight reduction and significant metabolic improvements [3]. However, postoperative outcomes vary widely among individuals. In recent years, there has been a growing interest in developing tools to predict the success of bariatric procedures in terms of weight loss and comorbidity remission.

The use of artificial intelligence (AI) and machine learning (ML) tools has gained significant relevance in medicine. In bariatric surgery, these technologies have proven useful in improving the prediction of postoperative complications, body mass index (BMI) progression, and the likelihood of remission of metabolic diseases such as type 2 diabetes mellitus [4]. Models, such as artificial neural networks, decision trees, and deep learning algorithms, have been implemented to assess surgical success based on preoperative variables and clinical follow-up [5]. ML applications in bariatric surgery have been explored for various purposes. For example, a Convolutional Neural Network (CNN)-based model to predict diabetes remission after gastric bypass, achieving over 80% accuracy [6]. In a previous study, the authors analyzed data from the Scandinavian Obesity Surgery Registry (SOReg), including 6542 patients, comparing Bayesian Network models (GBN) and Dynamic Bayesian Network (DBN) models to predict long-term health-related quality of life and the evolution of comorbidities after bariatric surgery [7]. This study found that DBN models were particularly effective, as they could incorporate temporal data to update predictions over time. Similarly, a multicenter study demonstrated that ML models improve their accuracy when incorporating follow-up data at 6 and 12 months postoperatively [8]. Additionally, explainable AI was explored for predicting body weight loss after sleeve gastrectomy, integrating both imaging and non-imaging medical data through a Wide and Deep Learning (WAD) approach [9].

Other studies have employed techniques such as Random Forest (RF), Support Vector Machines (SVMs), AdaBoost, Multilayer Perceptron (MLP), Convolutional Neural Networks (CNNs), Recurrent Neural Networks with Long Short-Term Memory (RNN_LSTM) from the TensorFlow library, and Transformer models to predict body mass index (BMI) progression up to five years after surgery [10,11]. One study also focuses on identifying factors associated with treatment dropout in patients with obesity and psychological disorders who are not candidates for bariatric surgery [12]. These studies highlight the potential of ML in this field. However, further validations in more diverse populations and improvements in model interpretability are necessary for effective clinical implementation.

The distinctiveness of this study lies in its exclusive use of preoperative variables—encompassing clinical, biochemical, and psychological factors—to predict body weight loss outcomes one year following sleeve gastrectomy. In contrast to previous studies that incorporate postoperative data or rely on large international datasets [5,6,7,8], our approach is grounded in a real-world clinical cohort, thereby providing locally relevant evidence and contributing a multifactorial and context-specific perspective to the field of bariatric surgery.

The main objective is to evaluate predictive variables for successful weight loss after Sleeve bariatric surgery using ML techniques, defining success as body weight loss exceeding 30%. By analyzing predictive models, this study aims to optimize patient selection and improve clinical decision-making. The following sections describe the methodology, present and discuss the results, and conclude with the main findings, limitations, and future research directions.

## 2. Materials and Methods

This section describes the dataset used in this study, including its origin and the selected variables. It details the data preprocessing steps, such as cleaning, transformation, and missing value imputation. Feature selection is performed to identify the most relevant variables. Finally, the machine learning algorithms evaluated for prediction are explained.

### 2.1. Dataset Description

This study is based on data collected between 2013 and 2018 from morbidly obese patients who underwent bariatric surgery. These patients were referred from the Endocrinology and Nutrition Department at the Clinical University Hospital of Valladolid (HCUV) to the General Surgery Department at Hospital Río Hortega (HURH) for surgery. Postoperative follow-ups were conducted at the Nutrition Section of the Endocrinology and Nutrition Department in the Eastern Area of Valladolid. All participants provided informed consent before inclusion in this study. This study was approved by the Ethics Committee of the Valladolid East Health Area (CEIm Valladolid Este), under approval number PI24621.

The collected data include clinical variables (patient identification, medical history, disease progression, and nutritional history), anthropometric variables (body measurements and bioimpedance analysis), biochemical markers, treatment-related variables and postoperative complications, and psychological and quality-of-life variables. These measurements were recorded at different times: before surgery and at 3, 6, and 12 months postoperatively.

The initial dataset, which contained 94 patients who had completed the third follow-up visit and had less than 50% missing data, was included in this study.

### 2.2. Variables

The objective of this study is to evaluate predictive variables for successful weight loss after Sleeve bariatric surgery, defining success as an excess weight loss greater than 30%. To develop the predictive model, preoperative variables were used as input features, while the target variable, Successfull_BodyWeight, was derived from the percentage of weight loss recorded at the third follow-up visit (12 months post-surgery).

The percentage of body weight loss (%PP) is calculated using the following equation:

(1) $$\%\mathrm{PP}=\frac{\text{Initial body weight}\;(\mathrm{Kg})-\text{Post-intervention body weight}\;(\mathrm{kg})}{\text{Initial body weight}\;(\mathrm{kg})}\;\times \;100$$

where initial body weight refers to the patient’s body weight before surgery, and post-intervention body weight corresponds to the weight recorded 12 months after surgery. To classify body weight loss success, the variable Successfull_BodyWeight was defined with two categories:

- 1 (Success): Patients who achieved a body weight loss greater than 30%.
- 0 (No success): Patients who did not reach this threshold.

From the 523 initial variables, a total of 95 input variables were selected, including both quantitative and qualitative variables. These were classified into different groups, as shown in Table 1.

### 2.3. Data Preprocessing

In this phase, an exploratory analysis was conducted to identify missing and outlier values. Variables corresponding to measurements taken at 3, 6, and 12 months post-surgery were removed, except for the percentage of weight loss at one year, which was used to calculate the target variable. Once this calculation was completed, the target variable was removed from the dataset to improve the model’s generalization capability.

To handle missing values, an imputation process was performed based on the variable type (categorical or numerical) and the amount of missing data. The K-Nearest Neighbors Imputer (KNN) method with k = 5 was applied to estimate missing values using the five closest neighbors for variables with more than 20% missing data. Variables with less than 20% missing values were imputed using the median, while categorical variables were imputed using the mode, following the SimpleImputer (strategy = “most_frequent”) method.

Categorical variables were converted into numerical values using Label Encoding and One-Hot Encoding techniques [13]. Label Encoding was applied to binary variables, converting their values into 0 and 1. For variables with more than two categories, One-Hot Encoding was used, creating new columns for each category type.

To examine the distribution of the variables, the Shapiro–Wilk test [14] was performed to determine which variables followed a normal distribution and which required transformation. This test provided a *p*-value for each variable, and *p* > 0.05 indicated a normal distribution, whereas *p* ≤ 0.05 led to the rejection of the normality hypothesis. Based on these *p*-values, normalization (MinMaxScaler) or standardization (StandardScaler) techniques were applied, depending on the machine learning algorithm used.

An imbalance in the target variable was identified, necessitating the application of the Synthetic Minority Over-sampling Technique (SMOTE) [15] to balance the classes by generating synthetic data for the minority class using the nearest neighbors approach.

To evaluate the models, the dataset was split into 70% for training and 30% for testing. The model was trained using the training set and later evaluated on the test set. Due to the high dimensionality of the dataset, Random Forest was applied to calculate feature importance, selecting the 10 most relevant variables while eliminating irrelevant information and mitigating overfitting.

Finally, data analysis and modeling were conducted on a system with Windows 11 (64-bit architecture), an Intel64 Family 6 Model 140 Stepping 2 (GenuineIntel) processor, and 15.68 GB of RAM. The programming language used was Python (version 3.12.3), with Jupyter Notebook (version 7.2.2) as the development environment. The pandas and numpy libraries were used for descriptive statistics and data transformation. Histograms and distribution plots were generated with matplotlib.pyplot and seaborn, while the pyreadstat library was employed to load the dataset in SPSS (version 29.0.2.0) (.sav) format.

### 2.4. Machine Learning Algorithms, Cross-Validation, and Model Evaluation

In this study, different machine learning algorithms were evaluated to identify the best predictive model for bariatric surgery success. The selection of these algorithms was based on previous research. The following models were implemented:

- Random Forest (RF): A supervised learning algorithm that combines multiple decision trees to produce a single prediction. It is efficient in analyzing complex, high-dimensional data, offers fast learning, and helps reduce overfitting [16].
- Support Vector Machine (SVM): This algorithm finds a hyperplane that separates classes in a high-dimensional space. It can be linear or nonlinear, depending on the selected kernel parameter [17].
- Multilayer Perceptron (MLP): A type of artificial neural network composed of multiple layers of neurons arranged sequentially. It includes an input layer, one or more hidden layers, and an output layer, all fully connected. During training, it adjusts connection weights using backpropagation to minimize errors [18].
- Extreme Gradient Boosting (XGBoost): An ensemble algorithm that applies the boosting techniques to enhance the performance of decision tree-based models. It focuses on minimizing errors through gradient reduction [19].
- Decision Tree (DT): A machine learning algorithm that splits data into branches based on feature-based rules. It allows for an intuitive visualization of the decision-making process [20].
- Logistic Regression (LR): A regression method used to predict the probability of a binary dependent variable. It applies a sigmoid function to transform the output into probabilities between 0 and 1 [21].

To improve hyperparameter selection, k-fold cross-validation was applied with k = 10 folds. The training set was divided into 10 subsets, where 9 folds were used for training and 1 fold for validation in each iteration. This process was repeated until each fold was used as a validation set. Hyperparameter optimization was performed by adjusting parameters specific to each ML model to enhance performance. The model evaluation was conducted using the following metrics: accuracy, recall, precision, F1-score, ROC-AUC, and confusion matrix.

## 3. Results

### 3.1. Dataset

This study analyzed a total of 94 patients with morbid obesity who underwent bariatric surgery and completed a one-year follow-up after the procedure. Among the studied patients, 68 were women and 26 were men.

Table 2 presents the results of various variables, comparing patients who achieved success and those who did not. For continuous variables, means ± standard deviations are reported, while for categorical variables, frequencies (%) are provided.

The results in Table 2 indicate that there is no significant difference between the two groups regarding age, height, and waist circumference, as reflected by *p*-values > 0.05. However, the BMI (*p* = 0.033) and preoperative body weight (*p* = 0.043) were higher in the successful group, while triglyceride levels were lower in this group. A difference was observed in diabetes mellitus prevalence, with fewer diabetic patients (16.1%) achieving body weight loss success. No significant differences were found in the remaining variables.

### 3.2. Predictor Variables and Class Balancing

**Figure 1:**
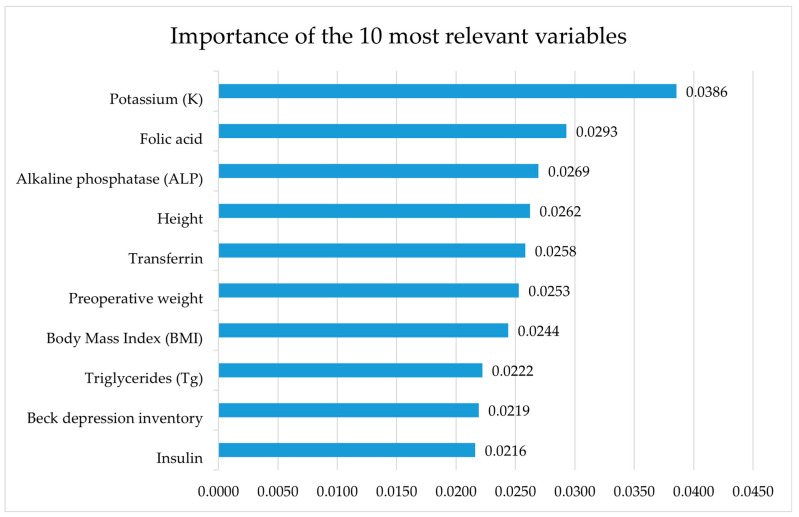
Importance of the 10 most relevant variables according to the Random Forest Classifier algorithm.

The dataset exhibited high dimensionality, prompting the use of the Random Forest Classifier to determine the importance of predictive variables. This method assesses variable importance based on the mean decrease in the Gini impurity index (IG). Figure 1 presents the 10 most important features selected by the algorithm.

The results indicate that among the biochemical variables, potassium (K) had the highest importance (0.0385), followed by folic acid (0.0293), alkaline phosphatase (0.0269), transferrin (0.0258), triglycerides (0.0222), and insulin (0.0216). Among the anthropometric variables, the most significant were height (0.0262), preoperative body weight (0.0253), and the BMI (0.0244). Regarding psychological and quality-of-life variables, the Beck depression inventory (0.0219) was the most relevant.

The target variable Successfull_BodyWeight showed an imbalance in class distribution, with 31 success cases and 63 non-success cases. To balance the classes, the SMOTE was applied to the training set, equalizing the distribution to 44 patients per class.

### 3.3. Model Evaluation

Six predictive models were developed: RF, MLP, XGBoost, DT, LR, and SVM. The best hyperparameters for each model were determined using optimization techniques such as RandomizedSearchCV and GridSearchCV. Table 3 presents the best-selected hyperparameters for each model.

A 10-fold cross-validation (cv = 10) was performed to evaluate the models, using the following metrics: accuracy, precision, recall, F1-score, and ROC-AUC. Table 4 presents the results of these performance metrics for each developed model.

The SVM algorithm achieved the best results, with a precision of 0.886, recall of 0.862, F1-score of 0.851, and AUC of 0.757, with a 95% CI between 0.5238 and 0.9658. The RF and MLP models had similar results, with equal accuracy and recall values and a slightly different precision and F1-score. However, MLP had a higher AUC (0.705) compared to RF (0.636). The XGBoost and DT models obtained the lowest scores, while LR showed intermediate results.

**Figure 2:**
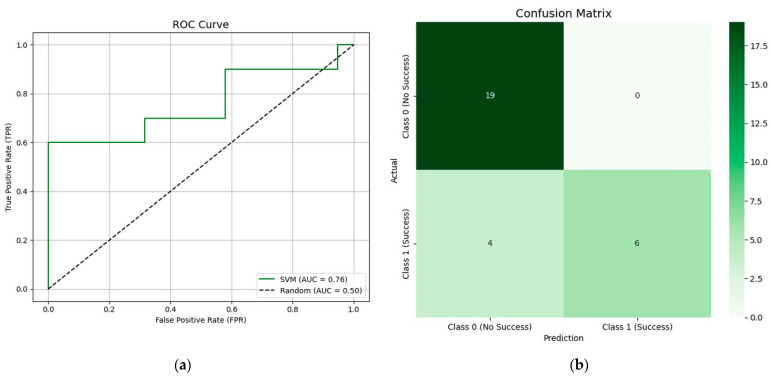
SVM model results: (**a**) ROC curve; (**b**) confusion matrix.

Figure 2a presents the ROC curve for the SVM model, which achieved the best results (AUC = 0.76). This ROC curve graphically represents the relationship between the true positive rate and the false positive rate across different threshold values. Figure 2b displays the confusion matrix, showing that the model correctly classified 19 non-success cases and 6 success cases, with 4 false negatives and 0 false positives.

## 4. Discussion

In this study, several machine learning models were applied to predict body weight loss success one year after Sleeve bariatric surgery, and the key predictive variables for success were identified. Although the primary objective was to evaluate the predictive variables, the different models were also compared to determine the best-performing one.

The evaluated models included RF, MLP, XGBoost, DT, LR, and SVM. Among these, SVM achieved the best performance, with an accuracy of 88% and an AUC of 0.76, with IC 95% between 0.5238 and 0.9658. This result aligns with previous studies that have used SVM for prediction in bariatric surgery. For example, a study [22] identified SVMs and neural networks as among the most effective ML approaches in bariatric surgery, particularly due to their capacity to handle complex variable interactions and generate accurate classifications.

Similarly, a study [10] applied various ML algorithms—including RF and SVM—to predict postoperative BMI in patients undergoing bariatric surgery. Their models, which incorporated both preoperative and longitudinal follow-up data, demonstrated strong predictive capabilities, especially when advanced ML techniques were employed. In contrast, our model achieved high predictive performance using preoperative variables alone, underscoring its applicability in early-stage clinical decision-making, when follow-up data are not yet available.

In addition, a study [2] proposed a machine learning model to predict body weight loss one year after Roux-en-Y gastric bypass. Their approach correctly classified a large proportion of unsuccessful cases but was less effective at identifying successful ones, with over 36% misclassification. Compared to this, our SVM model demonstrated better balance between groups, despite the use of a different surgical technique and a larger initial variable set.

Another study [8] on predictive models in bariatric surgery found that incorporating postoperative follow-up data from patient visits improved model performance. However, in the present study, the SVM demonstrated optimal performance even during the preoperative phase, highlighting its ability to identify patterns at this early stage.

Based on these findings, the most important variables for predicting body weight loss success after surgery were analyzed. Random Forest was used to select the 10 most relevant predictive variables, which was essential given the high dimensionality of the dataset. However, this method does not indicate whether a higher or lower value of a variable is associated with greater or lesser body weight loss.

The results showed that potassium (K) was the most important variable according to the Random Forest feature selection. Physiologically, potassium is a key electrolyte in energy metabolism and muscle function. Its deficiency has been linked to insulin resistance and cellular dysfunction, meaning low levels may indicate malnutrition or inadequate protein/energy intake before surgery, potentially affecting postoperative metabolic adaptation [23].

Folic acid was identified as the second most important variable, playing a key role in DNA synthesis, cell proliferation, and homocysteine metabolism. In obesity, folic acid levels may be reduced due to poor diet and altered liver metabolism. Folic acid deficiency has been linked to mitochondrial dysfunction and impaired energy metabolism [24].

The third most important variable was alkaline phosphatase. In obese patients, elevated levels may indicate liver dysfunction, such as non-alcoholic fatty liver disease, or bone disorders secondary to malnutrition [25].

Among anthropometric variables, height, preoperative body weight, and BMI were significant. Taller patients may have greater lean mass, leading to higher resting energy expenditure, which could facilitate weight loss after surgery. Higher initial body weight tends to result in greater absolute weight loss, though not necessarily a higher relative percentage of body weight loss. Moreover, a high BMI may indicate greater weight loss potential, but it is also associated with a higher risk of metabolic complications [26].

Transferrin, also identified as an important variable, is an iron-transporting protein and a marker of nutritional status and chronic inflammation. In obesity, its concentration may be altered due to systemic inflammation and insulin resistance. Low levels may indicate malnutrition or chronic inflammation, factors that can impact recovery and metabolic response after surgery [27].

Triglycerides reflect lipid metabolism and are often elevated in obesity and metabolic syndrome. The importance of this variable, along with statistical analysis, showed that patients who successfully lost weight had significantly lower triglyceride levels compared to those who did not. High triglyceride levels may indicate greater visceral fat reserves, which could influence the extent of weight loss [28]. In this context, preoperative insulin levels were identified as relevant predictors, potentially influencing metabolic response after surgery. Insulin plays a key role in energy metabolism regulation, and its relationship with insulin resistance in obese patients may affect the body’s ability to efficiently process nutrients after surgery [29].

The Beck depression inventory score was also a significant predictor, reinforcing the hypothesis that psychological factors can impact the success of bariatric surgery. Obesity is associated with higher depression rates, which may affect postoperative treatment adherence and eating behavior control. However, it was not possible to determine whether higher scores were associated with greater or lesser weight loss [30].

These findings highlight the importance of considering key medical factors in obesity and its post-bariatric surgery progression. The most important variables identified in this study are related to nutritional status, liver metabolism, metabolic state, psychological and behavioral factors, as well as structural and anthropometric aspects. Although these variables may influence surgical outcomes, their specific impact requires further analysis. Identifying these factors could help improve postoperative strategies, personalized treatments, and optimal follow-up care. Expanding this study to larger populations is necessary to validate the results, enhance prediction accuracy, and improve clinical application.

## 5. Limitations

This study presents several limitations that should be considered. The most significant limitation is the dataset size. Since it is relatively small, the model’s ability to generalize to larger samples may be affected. The analysis focused on patients from a single hospital, which may limit the model’s applicability to other patient populations with different characteristics. Although techniques were applied to minimize the impact of missing data imputation, biases may still be introduced, as the imputed values may not fully reflect the actual patient conditions. The clinical records were manually entered by healthcare personnel, which could lead to data entry errors. It is also important to note that ML algorithms detect patterns in data without considering clinical reasoning, relying solely on mathematical and statistical relationships among the available variables. Although variables obtained through bioimpedance were collected, they were not included in the final model due to the high percentage of missing data. Nevertheless, we acknowledge the clinical relevance of these measurements and suggest that future studies with more complete data should explore their predictive value in greater depth. Additionally, physical activity was not systematically recorded, which may have influenced the results and represents another limitation of this study. There are no records of actual food intake, which could be another limitation; however, the loss of weight and metabolic changes in these patients are mainly due to bariatric surgery, and all of them had received the same type of surgery by the same surgeon.

## 6. Conclusions

In this study, RF, MLP, XGBoost, DT, LR, and SVM algorithms were used to predict weight loss success after Sleeve bariatric surgery. A data preprocessing step was performed, where missing values were identified and imputed using KNN, median, and mode techniques. To balance the classes, SMOTE was applied to generate synthetic data. Due to the high dimensionality of the dataset, RF was used to identify the most important predictive variables. The best-performing model was the SVM.

The most relevant predictive variables were potassium (K), folic acid, alkaline phosphatase, height, transferrin, body weight, BMI, triglyceride (Tg), Beck depression inventory score, and insulin.

Future directions include the use of explainable AI techniques to analyze the impact of predictive variables. The development of a predictive calculator based on the models is proposed to assist in medical decision-making. Also, new ML models will be developed to evaluate predictive variables for the resolution of obesity-related comorbidities after Sleeve bariatric surgery, such as dyslipidemia, diabetes, and hypertension. Furthermore, models will be explored for predicting early and late surgical complications and postoperative hospital readmissions.

## Figures and Tables

**Table 1:** Description of input variables.

**Category**	**Variables**
Clinical and epidemiological	Sex, age. Hypertension (HTN), hypercholesterolemia, hypertriglyceridemia. Diabetes mellitus (DM). Alcohol and tobacco consumption. Hyperuricemia, steatosis, arthropathy, sleep apnea-hypopnea syndrome (SAHS). Multivitamins, adherence to multivitamins and medication, multivitamin change. Diuretics, statins. Systolic and diastolic blood pressure (SBP and DBP). Metabolic syndrome.
Biochemical	Blood glucose, urea, creatinine (Cr), insulin, uric acid. Glycated hemoglobin (HbA1C). Total cholesterol (TC), triglyceride (Tg), low-density lipoprotein (LDL), Glutamate-Oxaloacetate Transaminase (GOT), Glutamate-Pyruvate Transaminase (GPT), Gamma-Glutamyl Transferase (GGT), alkaline phosphatase (ALP). Total protein (TP), albumin, total bilirubin (TB), prealbumin. International Normalized Ratio (INR), fibrinogen. Sodium (Na), potassium (K), magnesium (Mg), calcium (Ca), phosphorus (P), parathyroid hormone (PTH). Vitamins A, E, D, K, B12, copper, zinc, folic acid, iron, ferritin, transferrin. Homeostatic Model Assessment (HOMA). Hemoglobin (Hb), high-density lipoprotein (HDL).
Anthropometric	Height, body weight, waist circumference. Body mass index (BMI), ideal weight, adjusted weight, and BMI classification.
Treatment-related and postoperative complications	Reintervention and cause, early and late complications, types of complications. Metformin, SGLT2 inhibitors, Glucagon-like peptide 1 (GLP1) agonists, inhibitors of the angiotensin-converting enzyme (ACE)/angiotensin receptor blockers (ARBs). Number of oral antidiabetic drugs (nº ADO), number of antihypertensive medications (nº HTm), number of lipid-lowering medications (nº cholesterol), number of preoperative medications.
Psychological and quality of life	Personality disorder (Salamanca questionnaire). Bulimia test (total scores, symptom scales, severity). Beck depression inventory. SF-36 test (overall SF-36 score, physical function, physical problems, pain, general health perception, vitality, social function, emotional problems, mental health, and time-related changes).

**Table 2:** Basal data analysis of surgery.

Variable	Success (n = 31)	Non-Success (n = 63)	*p*-Value
Age (years)	45.4 ± 8.0	45.8 ± 9.8	0.729
Height (cm)	166.2 ± 11.9	164.2 ± 10.2	0.357
Body mass index (BMI, kg/m^2^)	49.3 ± 6.4	46.4 ± 5.8	0.033
Body weight (kg)	137.0 ± 27.2	125.3 ± 22.0	0.043
Waist circumference (cm)	133.2 ± 12.5	129.7 ± 13.8	0.292
Blood glucose (mg/dL)	101.3 ± 11.6	115.3 ± 53.5	0.502
Glycated hemoglobin (HbA1C)	5.8 ± 0.4	6.1 ± 1.6	0.744
Homeostasis model assessment (HOMA)	7.1 ± 3.4	7.1 ± 4.0	0.464
Total cholesterol (TC) (mg/dL)	189.3 ± 30.0	185.9 ± 37.8	0.647
Triglycerides (Tg) (mg/dL)	125.0 ± 46.3	157.3 ± 74.8	0.012
Low-density lipoproteins (LDL) (mg/dL)	116.0 ± 27.3	110.5 ± 34.5	0.236
High-density lipoproteins (HDL) (mg/dL)	48.3 ± 11.3	46.5 ± 10.9	0.688
Bulimia test total score (points)	9.8 ± 5.6	9.7 ± 4.4	0.831
Beck depression inventory (points)	13.9 ± 8.7	11.1 ± 6.6	0.194
SF-36 test mean score (points)	47.9 ± 23.0	47.2 ± 24.8	0.866
Sex (male)	10 (32.3%)	16 (25.4%)	0.650
Hypertension (HTN)	16 (51.6%)	24 (38.1%)	0.306
Diabetes mellitus (DM)	5 (16.1%)	26 (41.3%)	0.028
Hypercholesterolemia	4 (12.9%)	16 (25.4%)	0.261
Hypertriglyceridemia	2 (6.5%)	4 (6.3%)	1.000

**Table 3:** Hyperparameter optimization for each model.

**Model**	**Hyperparameter Optimization**
RF	Number of trees: 200 Maximum tree depth: None (no limit) Minimum number of samples to split a node: 2 Class weights: {0:1, 1:5}
SVM	Kernel type: Radial Basis Function (RBF) Penalty parameter (C): 1 Gamma value (sensitivity of the model): 0.01 Class weights: {0:1, 1:3}
MLP	Hidden layer size: 100 neurons Initial learning rate: 0.1 Regularization parameter (alpha): 0.0001 Solver (optimizer): Adam
XGBoost	Number of trees: 200 Learning rate: 0.01 Maximum tree depth: 7 Positive class weight: 1 Subsample ratio: 1.0 Column subsample per tree: 0.8
DT	Maximum tree depth: 10 Class weights: balanced Minimum samples to split a node: 10 Minimum samples per leaf: 2 Maximum number of leaf nodes: None (no limit)
LR	Regularization parameter (C): 10 Class weights: {0:1, 1:1} Maximum iterations: 500 Data normalization method: RobustScaler

**Table 4:** Results of the metrics for each model.

Model	Metrics				
	Accuracy	Precision	Recall	F1-Score	AUC
RF	0.758621	0.767241	0.758621	0.732424	0.636842
MLP	0.758621	0.752575	0.758621	0.746153	0.705263
XGBoost	0.655172	0.617931	0.655172	0.604792	0.668421
DT	0.655172	0.628186	0.655172	0.628489	0.513158
LG	0.724138	0.714143	0.724138	0.702791	0.694737
SVM	0.862069	0.886057	0.862069	0.851396	0.757895

## Data Availability

The data used in this study are not publicly available due to privacy or ethical restrictions. They were obtained from the Nutrition Section of the Endocrinology and Nutrition Department in the Eastern Area of Valladolid and are available from the corresponding author upon reasonable request and with permission from the data provider.

## Abbreviations

BMI	Body Mass Index
ML	Machine Learning
AI	Artificial Intelligence
RF	Random Forest
SVM	Support Vector Machine
MLP	Multilayer Perceptron
XGBoost	Extreme Gradient Boosting
DT	Decision Tree
LR	Logistic Regression
CNN	Convolutional Neural Network
RNN_LSTM	Recurrent Neural Network with Long Short-Term Memory
WAD	Wide and Deep Learning
SMOTE	Synthetic Minority Over-sampling Technique
ROC-AUC	Receiver Operating Characteristic Area Under Curve
